# Activation of Oncogenic and Immune-Response Pathways Is Linked to Disease-Specific Survival in Merkel Cell Carcinoma

**DOI:** 10.3390/cancers14153591

**Published:** 2022-07-23

**Authors:** Benjamin Sundqvist, Sami Kilpinen, Tom Böhling, Virve Koljonen, Harri Sihto

**Affiliations:** 1 Department of Pathology, University of Helsinki and Helsinki University Hospital, 00014 Helsinki, Finland; tom.bohling@helsinki.fi (T.B.); harri.sihto@helsinki.fi (H.S.); 2 Molecular and Integrative Biosciences Research Programme, University of Helsinki, 00014 Helsinki, Finland; sami.kilpinen@helsinki.fi; 3 Department of Plastic Surgery, University of Helsinki and Helsinki University Hospital, 00280 Helsinki, Finland; virve.koljonen@helsinki.fi

**Keywords:** Merkel cell carcinoma, transcriptome, signaling pathway, survival, gene expression

## Abstract

**Simple Summary:**

Merkel cell carcinoma (MCC) is a rare and aggressive skin cancer. Developing targeted therapies for MCC requires increased understanding of the mechanisms driving tumor progression. In this study, we aimed to identify genes, signaling pathways, and processes that play crucial roles in determining disease-specific survival in MCC. We analyzed the gene expression of 102 MCC tumors and identified genes that were upregulated among survivors and in patients who died from MCC. We cross-referenced these genes with online databases to identify the pathways and processes in which they function. Genes upregulated among survivors were mostly immune response related and genes upregulated among patients who died from MCC function in various pathways that promote cancer progression. These results could guide future studies investigating whether these genes and pathways could be used as prognostic markers, as markers to guide therapy selection, or as targets of precision therapy in MCC.

**Abstract:**

Background: Merkel cell carcinoma (MCC) is a rare but highly aggressive neuroendocrine carcinoma of the skin with a poor prognosis. Improving the prognosis of MCC by means of targeted therapies requires further understanding of the mechanisms that drive tumor progression. In this study, we aimed to identify the genes, processes, and pathways that play the most crucial roles in determining MCC outcomes. Methods: We investigated transcriptomes generated by RNA sequencing of formalin-fixed paraffin-embedded tissue samples of 102 MCC patients and identified the genes that were upregulated among survivors and in patients who died from MCC. We subsequently cross-referenced these genes with online databases to investigate the functions and pathways they represent. We further investigated differential gene expression based on viral status in patients who died from MCC. Results: We found several novel genes associated with MCC-specific survival. Genes upregulated in patients who died from MCC were most notably associated with angiogenesis and the PI3K-Akt and MAPK pathways; their expression predominantly had no association with viral status in patients who died from MCC. Genes upregulated among survivors were largely associated with antigen presentation and immune response. Conclusion: This outcome-based discrepancy in gene expression suggests that these pathways and processes likely play crucial roles in determining MCC outcomes.

## 1. Introduction

Merkel cell carcinoma (MCC) is a neuroendocrine carcinoma of the skin with a poor prognosis. The survival rate of MCC varies significantly; population-based studies from New Zealand, Finland, and the United States revealed 5-year disease-specific survival rates of 45%, 59%, and 73%, respectively [1,2,3]. In most MCCs (approximately 80% of MCC tumors in the northern hemisphere), the genome of Merkel cell polyomavirus (MCPyV) is integrated in the tumor cell genome [4,5]. Our group and others have previously identified several clinicopathological factors that negatively influence survival in MCC, such as tumor MCPyV-negativity, lack of tumor-infiltrating lymphocytes, male sex, larger primary tumor size, presence of lymph node or systemic metastasis at diagnosis, and immunosuppression [1,2,3,4,6,7,8,9,10,11]. Despite the generally poor survival of MCC, there is a significant proportion of patients in our Finnish population-based cohort who are still alive over a decade after the initial diagnosis. The treatment of MCC generally consists of surgical removal accompanied by sentinel lymph node biopsy and potential lymph node evacuation followed by radiotherapy. In disseminated MCC, immune-checkpoint inhibitors, namely PD-1 or PD-L1 inhibitors, are frequently used [12,13]. To improve the poor prognosis of MCC by means of developing effective targeted therapies, an improved understanding of the mechanisms that drive tumor progression is necessary.

In a recent study by Harms et al., targeted DNA and transcriptional profiling of 63 and 26 pre-defined, cancer-relevant genes, respectively, was performed. The study revealed that oncogene-activating mutations in PIK3CA, IDH2, and JAK2 were associated with poorer disease-specific survival in MCPyV-positive cases. Improved disease-specific survival was associated with higher expression of the pro-inflammatory transcripts IDO1, IFNG, and GZMA in MCPyV-positive cases, whereas high expression of the oncogene transcripts BRAF, RET, and UBE2C was associated with poorer survival in MCPyV-negative cases [10]. A previous transcriptomic study by Paulson et al. revealed that tumors from patients with a good prognosis exhibited overexpression of immune response-associated genes, particularly genes associated with cytotoxic CD8+ lymphocytes. However, genes overexpressed in tumors from patients with a poor prognosis were not examined in this study [11].

In this study, we aimed to identify genes, processes, and pathways that play crucial roles in causing MCC-specific death and MCC-specific survival that may be targeted in the treatment of MCC or may be used in guiding therapy selection or as prognostic markers. We investigated transcriptomes from the primary MCC tumors of 102 patients to identify the genes that were most significantly upregulated among survivors and among patients who died from MCC. We subsequently cross-referenced these genes with gene ontology and pathway databases to identify the biological processes and signaling pathways associated with these differentially expressed genes.

## 2. Materials and Methods

### 2.1. Patients, Clinical Data, and Tissue Samples

Data on patients diagnosed with MCC in Finland from 1983 to 2013 were obtained from the Finnish Cancer Registry and Helsinki University Hospital files. Clinical details were extracted from hospital records. Formalin-fixed, paraffin-embedded (FFPE) tissue blocks from primary tumors were retrieved from the pathology archives. MCC diagnoses were confirmed in a blinded fashion from our earlier studies according to well-established criteria by two researchers with special expertise in MCC pathology [14]. The study protocol was approved by the Ethics Committee of Helsinki University Central Hospital. The Ministry of Health and Social Affairs granted permission to collect patient data and the National Authority for Medicolegal Affairs to collect tissue samples.

The patients were subdivided into the following groups: the poor prognosis group (patients who died from MCC) and the good prognosis group (patients who were either alive at the most recent follow-up or died from a cause unrelated to MCC). The genes that were upregulated in the tumors of patients in the poor prognosis group when compared with the good prognosis group will be referred to as death-associated genes (DAGs); genes that were upregulated in tumors of patients in the good prognosis group when compared with the poor prognosis group will be referred to as survival-associated genes (SAGs).

MCPyV detection from paraffinized tumor blocks was performed previously and is described in detail elsewhere [4]. Briefly, the presence of MCPyV DNA was analyzed from DNA extracted from representative deparaffinized tumor sections. Quantitation of MCPyV DNA was performed using real-time polymerase chain reaction (PCR). The relative DNA sequence copy number for each tissue sample was expressed as a ratio of MCPyV DNA to protein tyrosine phosphatase gamma receptor gene DNA. The sample was considered positive when MCPyV DNA copy number per reference gene was greater than 0.1.

### 2.2. RNA Extraction from FFPE Samples

RNA extraction and sequencing were performed on FFPE primary tumor samples from 120 patients from whom sufficient representative tumor tissue was available. Fourteen patients were excluded from the study as the corresponding samples did not pass the quality control during the processing of sequencing data. A further four patients were excluded because their tumor MCPyV status was not known. The final sample size was 102 patients.

Two 10 µM sections from FFPE MCC samples of a cancer-representative area were used as starting material for the RNA extractions. RNA extraction was performed with a QIASymphony SP instrument (QIAGEN GmbH, Hilden, Germany) and QIAsymphony RNA extraction kit (cat. No: 931636, QIAGEN GmbH, Hilden, Germany), following the manufacturer’s protocol. The quality and quantity of the extracted RNA were measured using a 2100 Bioanalyzer (Agilent technologies, Santa Clara, CA, USA). The average RNA integrity number (RIN) was 2.1.

### 2.3. 3′ RNA Sequencing

RNA sequencing was performed by the sequencing unit of the Institute for Molecular Medicine Finland (FIMM) Technology Centre, University of Helsinki. Prior to library preparation, 1 µL of 1:1000 ERCC RNA spike-in control was added to each sample. A QuantSeq 3′ mRNA-Seq Library kit (Lexogen, Vienna, Austria, version 015UG009V0221) was used to prepare the RNA sequencing library in 48 sample batches. The library preparation was performed according to the manufacturer’s instructions and is described in detail elsewhere [15]. The sample libraries were homogenous in average size and concentration. The sample libraries were pooled together by their molarity for the sequencing with 39 samples per lane. A HiSeq 2500 instrument from Illumina was used as the sequencer with high-output mode and v4 chemistry. The sequencing run was paired-end with a read length of 101 bp and one mismatch allowed in demultiplexing. The RNA sequence data were submitted to the SRA database in NCBI and can be accessed under the BioProject PRJNA775071.

#### Processing of Sequencing Data

Htseq-count files from Bluebee sequencing process were read into R [16] (version 4.1.0) and were matched to clinical data per sample. Gene annotation was retrieved from AnnotationHub (snapshotDate: 20 October 2021). Data were imported into DGEList object using edgeR package [17] (version 3.36.0). Genes were further filtered by filterByExpr function (default parameters) from edgeR. log2 counts per million were calculated with cpm function (default parameters) from edgeR. Additional sample quality control was performed by excluding samples with median logcount with deviation more than 10% from the median logcount over all samples. Samples without MCPyV status in clinical data were excluded as the last step of sample-based filtering.

In order to validate that our transcriptomic data derived from FFPE samples are comparable to data from fresh frozen tissues, we performed a comparative analysis between the data used in this study and data from Harms et al. (GSE39612) [18]. Data from Harms et al. were retrieved from GEO as GSE matrix with log2 RMA normalized signal intensity per sample per Affymetrix probeset. Intensities were median merged per gene symbol as per GPL data provided by GEO for the study in question. Only data from tumors with a known MCPyV status were included. We then performed a differential expression test of genes between MCPyV-positive and MCPyV-negative tumors separately in GSE39612 data and FFPE derived data. In the case of FFPE data, this was done with DESeq2 R package, and for GSE39612, it was done with limma R package. Of the top 250 (based on B-statistics) differentially expressed genes from the analysis of GSE39612, we further analyzed the 136 genes that were present in the FFPE dataset. LogFC-values of these 136 genes between MCPyV-positive and MCPyV-negative tumors in both datasets were plotted. The correlation between the logFC values of these 136 genes in the two datasets was calculated with the sm_statCorr() function in R with Pearson as the correlation metric.

### 2.4. Identification of DAGs and SAGs

A differential expression test of genes between the good and poor prognosis groups was performed using estimateDisp and exactTest function from edgeR with default parameters. DAGs were defined as those with a positive logFC with Benjamini–Hochberg (BH) corrected *p*-value < 0.05; these were genes found to be upregulated in samples of the poor prognosis group. SAGs had a negative logFC with BH corrected *p*-value < 0.05; these were genes found to be upregulated in samples of the good prognosis group. Altogether, we identified 79 genes differentially expressed between patients in these two groups.

### 2.5. Clustered Heatmap of Differentially Expressed Genes

To visualize the gene expression patterns of these 79 identified genes, we drew a conventional heatmap with 102 patients on the *x*-axis and the 79 genes on the *y*-axis. Both axes were clustered using 1-Pearson correlation coefficient as the distance measure and the linkage method Ward.D2. The rows of the matrix were scaled to have a mean of 0 and a standard deviation of 1.

### 2.6. Gene Ontology and Signaling Pathway Analyses of DAGs and SAGs

The gene ontology and signaling pathway analyses of DAGs and SAGs were performed by separately cross-referencing the lists of DAGs and SAGs with the Gene Ontology Biological Process (GO BP), the Gene Ontology Molecular Function (GO MF), and the Kyoto Encyclopedia of Genes and Genomes (KEGG) Pathway databases using Enrichr [19,20]. Enrichr is a publicly available gene set search engine (http://amp.pharm.mssm.edu/Enrichr, accessed on 31 December 2021) [21]. The enriched GO terms and KEGG pathways were subsequently ranked by significance according to their *p*-values.

### 2.7. Statistical Analysis

Statistical analysis was performed with SPSS statistics 26.0 software (IBM Corporation, New York, NY, USA). The MCC-specific prognostic effects of tumor MCPyV status, sex, age at the time of diagnosis, tumor location, and the presence of lymph node or systemic metastasis at diagnosis were assessed by Kaplan–Meier analysis and logrank test. The factors that were significantly associated with MCC-specific survival in univariate analyses were included in a Cox regression multivariate analysis. In both uni- and multivariate survival analyses, MCC-specific survival was calculated from the date of diagnosis to death considered to be due to MCC, censoring subjects alive on their last follow-up date, and subjects who died from a cause unrelated to MCC. The correlation between the abundance of tumor-infiltrating, CD3-positive lymphocytes and the expression levels of the genes CD74, TAP2, PSME1, HLA-DRA, and HLA-E was investigated by calculating Pearson’s product–moment correlation in each case. The abundance of tumor-infiltrating lymphocytes was investigated previously and is described in detail elsewhere; data were available in 80/102 cases [6].

### 2.8. Survival of ≥3 Years as Inclusion Criterion for the Good Prognosis Group

In order to evaluate the consistency of our findings, we repeated the differential expression test of genes between the good and poor prognosis group, this time only including the 40 patients who survived at least 3 years after initial diagnosis in the good prognosis group. We subsequently cross-referenced the differentially expressed genes yielded by this control analysis with the GO and KEGG databases in the manner described in Section 2.6.

### 2.9. Differential Expression Test of DAGs Based on MCPyV Status

In order to investigate potential relationships between DAG-expression and negative MCPyV status, we repeated the differential expression test of genes between MCPyV-positive (*n* = 15) and MCPyV-negative (*n* = 13) tumors of patients who died from MCC.

## 3. Results

### 3.1. Overview of Patients

Detailed patient clinical data are shown in Table 1. MCC-specific death occurred in 28/102 (27%) cases and occurred within 5 years of initial MCC diagnosis in 25/28 (89%) cases. The median survival time before MCC-specific death was 2.3 years (range 0.3 to 14.8 years). There was one extreme outlier who survived 14.8 years before MCC-specific death; the second longest survival time was 6.3 years. The median follow-up time before death from a cause unrelated to MCC, or until the last follow-up date at which the patient was still alive, was 4.4 years (range 0.04 to 33.2 years). Kaplan–Meier analysis revealed that MCPyV-negativity, male sex, and the presence of lymph node or systemic metastasis at diagnosis correlated with poorer MCC-specific survival (all *p* < 0.001). Table 2 shows the results of the Cox regression multivariate analysis; all three variables were significantly associated with poorer MCC-specific survival in the multivariate analysis.

### 3.2. Identification of DAGs and SAGs

**Figure 1:**
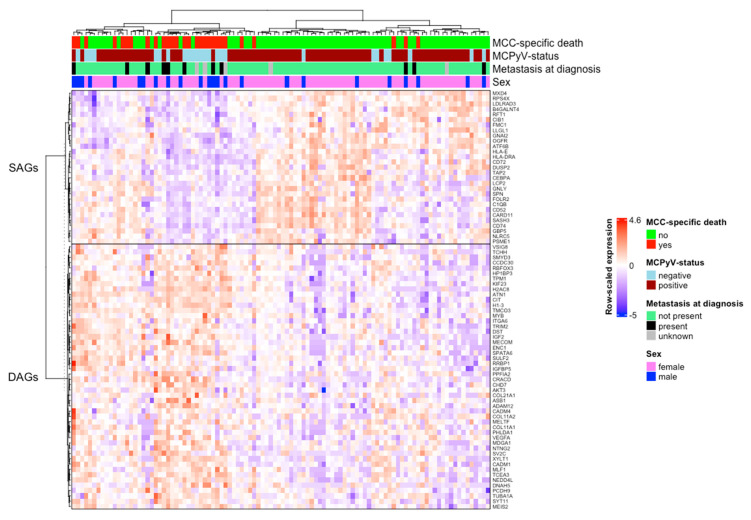
Death- and survival-associated gene expression across 102 MCC samples. The heatmap illustrates the expression of death- and survival-associated genes (DAGs and SAGs) across all samples. On the left is a phylogram of the 79 differentially expressed genes based on their expression across the samples. The upper main branch represents SAGs and the lower main branch represents DAGs. On the right are the gene symbols. At the top is a phylogram of the samples based on their expression of DAGs and SAGs. The samples from the poor prognosis group are depicted in red and the samples from the good prognosis group are depicted in green.

The differential expression test of genes revealed a total of 50 DAGs and 29 SAGs with a BH corrected *p*-value < 0.05. A summary of the upregulated genes, along with their corresponding logFC (fold change) values, false detection rates (FDR), and *p*-values, can be found in Table 3. A heatmap illustrating the expression of DAGs and SAGs across all samples can be found in Figure 1.

### 3.3. GO Enrichment and KEGG Signaling Pathway Analysis of DAGs and SAGs

The top 10 GO BP and GO MF terms most significantly enriched by the DAGs and SAGs, respectively, can be found arranged according to their *p*-values in Table 4 and Table 5, along with their corresponding q-values and a list of the specific genes causing the enrichment. The q-value is an adjusted *p*-value calculated using the BH method for correction for multiple hypotheses testing. The top 10 KEGG pathway terms most significantly enriched by DAGs and SAGs can be found in the same format in Table 6.

Cancer-relevant processes, functions, and pathways enriched by the DAGs included the PI3K-Akt signaling pathway, the MAPK signaling pathway, and angiogenic processes. Considering angiogenesis, the most significantly enriched GO terms included regulation of vascular associated smooth muscle cell migration, positive regulation of vascular endothelial cell proliferation, vascular endothelial growth factor receptor 2 binding, and vascular endothelial growth factor receptor binding. Among the most significantly enriched KEGG pathway terms was VEGF signaling pathway and among the DAGs was vascular endothelial growth factor A (VEGFA). Five of the DAGs were associated with the KEGG pathway term PI3K-Akt signaling pathway (MYB, AKT3, IGF2, ITGA6, and VEGFA) and four of the DAGs were associated with the KEGG pathway term MAPK signaling pathway (MECOM, AKT3, IGF2, and VEGFA).

The DAGs also exhibited enrichment of developmental pathways and processes, such as the GO PB terms chordate embryonic development (CHD7, IGF2, XYLT1, VEGFA, SULF2), in utero embryonic development (CHD7, IGF2, VEGFA), and skeletal system development (CHD7, COL11A2, IGF2, XYLT1, SULF2).

The processes, functions, and pathways most significantly enriched by the SAGs were mostly related to immune response, particularly to antigen processing and MHC-dependent antigen presentation, and to T-cell mediated immune response. Examples include the KEGG pathway term antigen processing and presentation (CD74, TAP2, PSME1, HLA-DRA, HLA-E) and the GO BP terms T cell receptor signaling pathway (PSME1, HLA-DRA, LCP2, CARD11) and positive regulation of lymphocyte proliferation (SASH3, CD74, HLA-E).

Complete lists of all the GO BP, GO MF, and KEGG pathway terms that had *p*-values of <0.05, enriched by the DAGs and SAGs, are presented in Supplementary Tables S1 and S2.

### 3.4. Survival of ≥3 Years as Inclusion Criterion for the Good Prognosis Group

Including only patients who were still alive 3 years after diagnosis in the good prognosis group resulted in an increased amount of differentially expressed genes. The number of genes upregulated in the good prognosis group increased to 82, including 22/29 of the original SAGs; the number of genes upregulated in the poor prognosis group increased to 118, including 40/50 of the original DAGs. Considering the SAGs of this control analysis, the vast majority of the most significantly enriched GO and KEGG pathway terms were still immune response-related, especially related to antigen processing and MHC-dependent antigen presentation. The GO and KEGG pathway terms most significantly enriched by the DAGs in this control analysis still included embryonic developmental processes. The KEGG pathways PI3K-Akt and MAPK signaling pathway were still enriched by the same DAGs, with the exception of FGFR2 instead of VEGFA in both cases; however, the *p*-values were no longer significant owing to the increased number of differentially expressed genes. The DAGs in this control analysis also exhibited enrichment of several terms related to mitosis as well as chromatin binding, the latter in part caused by the inclusion of several genes encoding histone proteins; the main finding that was lost in this control analysis was the DAG VEGFA, and thereby several GO and KEGG pathway terms related to angiogenesis. Complete lists of the differentially expressed genes yielded by this control analysis, as well as the GO and KEGG pathway terms significantly enriched by them, are provided in Supplementary Tables S3–S5.

### 3.5. Differential Expression Test of DAGs Based on MCPyV Status

The differential expression test of genes between MCPyV-positive and MCPyV-negative tumors of patients who died from MCC yielded 100 genes significantly upregulated in MCPyV-negative tumors. Of these genes, eight (VSIG8, NEDD4L, ITGA6, KIF23, IGF2, H1-3, DST, COL21A1) were among the DAGs. A complete list of these 100 genes is provided in Supplementary Table S6.

### 3.6. Correlation between Tumor-Infiltrating Lymphocytes and SAGs Related to Antigen Processing and Presentation

The correlation analysis between an abundance of CD3-positive, tumor-infiltrating lymphocytes and the expression levels of the five SAGs causing the enrichment of the KEGG pathway antigen processing and presentation (CD74, TAP2, PSME1, HLA-DRA, and HLA-E) revealed a significant and positive Pearson’s product–moment correlation in each case. Detailed results are presented in Supplementary Table S7.

### 3.7. Comparison of FFPE Data to Data from Fresh Frozen Tissues

Out of the 250 genes most significantly differentially expressed between MCPyV-positive and MCPyV-negative tumors in GSE39612, 136 genes were present in our FFPE data. The correlation analysis between the logFC-values of these 136 genes based of MCPyV status in GSE39612 and our data yielded a Pearson correlation coefficient of 0.82 (*p* < 0.001). A list of these 136 genes, together with their logFC-values in GSE39612 and our data, as well as a scatter plot of their expression, is provided in Supplementary Table S8.

## 4. Discussion

We found a dichotomous gene expression profile between the tumors of the poor and good survival groups. Many of the DAGs, which were upregulated in the poor survival group, function in various oncogenic pathways and processes. The SAGs, which were upregulated in the good survival group, were to a large extent immune-response-related genes.

We found a clear association of DAGs with angiogenesis. Angiogenesis plays a crucial role in the progression of solid tumors, and increased tumor vascularization is a factor predicting poor prognosis in MCC [22]. VEGFA, a proangiogenic growth factor that was among the DAGs, has been found to be expressed in the majority of MCC tumors based on immunohistochemistry results. Overexpression of VEGFA has been shown to correlate with metastatic tumor spread of MCC [23,24].

Five of the DAGs were associated with the KEGG pathway term PI3K-Akt signaling pathway, and four of the DAGs were associated with the KEGG pathway term MAPK signaling pathway. Furthermore, although not recognized in the KEGG pathway database, the DAGs SULF2, RBFOX3, and COL11A1 have been reported to upregulate the PI3K-Akt signaling pathway and the DAGs ITGA6, SMYD3, and ENC1 have been reported to upregulate the MAPK signaling pathway in other malignancies [25,26,27,28,29,30,31]. MCPyV small tumor antigen has previously been demonstrated to activate p38 MAPK signaling in MCC, and immunohistochemical findings have demonstrated high degrees of activating AKT phosphorylation in MCC [32,33]. The PI3K-Akt and MAPK signaling pathways both serve oncogenic roles in several malignancies, such as stimulating cellular proliferation, inhibiting apoptosis, promoting tumor invasion and metastasis, and stimulating angiogenesis, which in the case of the MAPK pathway is partially mediated by upregulation of VEGFA [34,35].

Among the DAGs were also insulin-like growth factor 2 (IGF2), a protumorigenic growth factor, and IGFBP5, which can either inhibit or potentiate insulin-like growth factor-signaling depending on the context [36]. Insulin-like growth factor binding, insulin-like growth factor I binding, and insulin-like growth factor II binding were among the most enriched GO MF terms. MCC has previously been shown to express insulin-like growth factor-I receptor, but to the best of our knowledge, insulin-like growth factor signaling in MCC has not otherwise been reported [37].

Considering clinical implications, the aforementioned pathways and processes include several potentially viable targets for pharmacological intervention. Kervarrec et al. suggested that VEGFA may be a potential therapeutic target in MCC following promising results from drug trials in mouse models using the monoclonal antibody bevacizumab [38]. The PI3K-Akt and the MAPK signaling pathways are two well-known oncogenic pathways, for which there are numerous established methods of pharmacological inhibition used in the treatment of other malignancies. These include PI3K, Akt, and mTOR inhibitors for the PI3K-Akt pathway and BRAF and MEK inhibitors for the MAPK pathway [39,40]. Furthermore, the insulin-like growth factor pathway constitutes another oncogenic pathway that can be targeted by blocking the IGF-1 receptor or its ligands IGF-1 and IGF-2 [41].

Other notable DAGs included MYB, DST, and KIF23. MYB (c-MYB) encodes an oncogenic transcription factor that regulates processes such as cell proliferation and apoptosis in several other malignancies. Small-molecule inhibitors of c-MYB, such as celastrol and blumbagin, have shown promising results in cell cultures and mouse models [42]. DST encodes a barrier protein that supports melanoma cell growth in vitro and in vivo, likely by interfering with immune cell infiltration or by enhancing angiogenesis [43]. KIF23 encodes a microtubule-associated motor protein involved in the regulation of cytokinesis. Upregulation of KIF23 increases cell proliferation and worsens prognosis in other malignancies, such as gastric cancer. Knockdown of KIF23 resulted in marked inhibition of proliferation in gastric cancer [44]. Other DAGs, the silencing of which has been demonstrated to inhibit cell proliferation or increase apoptosis in other malignancies, include MLF1, MELTF, CIT, RRBP1, CHD7, and MEIS2 [45,46,47,48,49,50].

Another curious finding considering DAGs was enrichment of developmental pathways and processes. This may suggest that the cancer cells of more aggressive MCC revert to a more primitive, stem-cell-like state. An embryonic stem-cell-like gene expression pattern has been found to correlate with poor tumor differentiation and poor prognosis in other malignancies [51]. So-called cancer stem cells that bear specific cell-surface markers and possess the abilities of self-renewal, induction of metastasis, evasion of apoptosis, and resistance to conventional cancer treatments have been described in several other malignancies, but have not as of yet been characterized in MCC. Advances have been made in specifically targeting these cells, including immunotherapy and gene therapy [52].

For SAGs, we found a clear association with pathways and processes related to immune response. Almost all of the most significantly enriched GO BP, GO MF, and KEGG pathway terms were immune-response-related and were especially related to antigen processing, MHC-dependent antigen presentation, and T-cell mediated immune response. These findings underline the importance of a functional immune system, capable of creating a hostile tumor microenvironment, for MCC-specific survival. These findings are also consistent with previous findings on the abundance of tumor-infiltrating lymphocytes, notably CD8-positive T-cells, as a strong predictor of good survival in MCC [6,8,9,11].

The positive correlation between the abundance of CD3-positive, tumor-infiltrating lymphocytes and the expression levels of the SAGs causing the enrichment of the KEGG pathway antigen processing and presentation suggests that the abundance of CD3-positive lymphocytes functions as a surrogate marker for an immunogenic gene expression signature.

Notable SAGs involved in processes not related to immune response included DUSP2, LLGL1, STAT1, and OGFR. DUSP2 encodes a phosphatase that deactivates protumorigenic MAP-kinases, the loss of which predicts a poor prognosis in bladder cancer [53]. LLGL1 encodes a cytoskeleton-associated protein involved in maintaining cell polarity; the loss of LLGL1 is associated with a loss of cellular adhesion, dissemination of cells, and distant metastases in several cancers including gastric cancer and malignant melanoma [54,55]. STAT1 encodes a protein that serves tumor-suppressive functions in many cancers and has been recognized as a potential biomarker for patient selection for treatment with anti-PD-1/anti-PD-L1 antibodies in breast cancer, as p-STAT1 correlates with higher PD-L1 and HLA class I expression [56,57]. Upregulation of opioid growth factor signaling through OGFR (opioid growth factor receptor) suppresses proliferation in several other malignancies, including lung and ovarian cancer [58,59].

Owing to the strong correlation between MCPyV-negativity and MCC-specific death, we repeated the differential expression test of genes based on MCPyV status within the poor prognosis group. Of the 50 DAGs, eight were significantly upregulated in MCPyV-negative tumors, suggesting that their prognostic relevance resulted at least in part from an association with MCPyV-negativity. The remaining 42 DAGs were not significantly differentially expressed based on MCPyV status within the poor prognosis group, suggesting a prognostic relevance regardless of viral status.

It should be noted that the average quality of the extracted RNA was fairly poor (average RIN of 2.1), as is often the case with FFPE samples. However, it has been shown that 3′ tag counting, such as that used in this study, markedly decreases the amount of false positives when studying differential expression of genes in samples of varying RIN at the expense of decreased sensitivity [60]. This study utilized a sequencing pipeline optimized for FFPE samples. Specifically, QuantSeq sequences only the 3′ end of the RNA transcript, thus significantly reducing the impact of partial RNA fragmentation. This is in contrast to, for example, Illumina’s TruSeq, which sequences most of the transcript. In a study comparing Lexogen’s QuantSeq and Illumina’s TruSeq, there was a strong correlation between the methods concerning the average expression values for all expressed genes. Both methods identified a similar number of expressed protein-coding genes, with QuantSeq identifying approximately 94% of the protein-coding genes found by TruSeq [15]. The correlation analysis between GSE39612 and our transcriptomic data revealed a strong correlation between the logFC-values based on the MCPyV status of the 136 genes studied. This suggests a high specificity of our FFPE data as compared with data from fresh frozen tissues when studying differentially expressed genes. Another challenge with the study design is that, because we analyzed bulk transcriptomic information, we cannot say if a certain expression signature originates from a specific subset of cells in the tumor or if it represents gene expression of the tumor overall.

## 5. Conclusions

In summary, we found several novel genes associated with disease-specific survival in MCC. The DAGs were most notably associated with angiogenesis, the PI3K-Akt signaling pathway, the MAPK signaling pathway, and embryonic developmental processes. The SAGs were most notably associated with antigen presentation and immune response. Further studies are required to determine if some of these genes could be used clinically as prognostic markers, as markers to guide therapy selection, or as targets of precision therapy in MCC.

## Figures and Tables

**Table 1:** Clinicopathological features of patients.

Characteristic	*n* = 102 (%)
Sex	
Male	24 (24)
Female	78 (76)
Age	Range 46–100
≤50 years	2 (2.0)
51–69 years	19 (19)
70–84 years	52 (51)
85–100 years	29 (28)
Died from MCC	
Yes	28 (27)
No	74 (73)
Tumor location	
Head and neck	64 (63)
Torso	10 (10)
Limbs	28 (27)
Stage ^1^ at diagnosis	
I	49 (51)
II	24 (26)
III	8 (8.5)
IV	3 (3.2)
Data not available	8
Metastasis ^2^ at diagnosis	
Present	11 (11)
Not present	86 (89)
Data not available	5
MCPyV status	
Negative	27 (26)
Positive	75 (74)

^1^ American Joint Committee on Cancer classification for Merkel cell carcinoma, eighth edition. ^2^ Lymph node or systemic. MCPyV = Merkel cell polyomavirus.

**Table 2:** Cox regression multivariate analysis of MCC-specific survival.

Variable	HR (95% CI)	*p*-Value
MCPyV-negativity	2.90 (1.28–6.60)	0.011
Metastasis ^1^ at diagnosis	7.78 (3.24–18.70)	<0.001
Male sex	3.04 (1.37–6.73)	0.006

^1^ Lymph node or systemic. HR = hazard ratio, CI = confidence interval.

**Table 3:** Genes associated with disease-specific death and survival in a series of 102 Merkel cell carcinoma patients.

Death-Associated Genes				Survival-Associated Genes			
Gene	logFC	*p*-Value	FDR	Gene	logFC	*p*-Value	FDR
TCHH	2.11	8.07 × 10^6^	0.010	GNLY	−2.00	2.71 × 10^5^	0.017
IGF2	1.90	2.03 × 10^5^	0.014	CEBPA	−1.82	5.43 × 10^5^	0.022
DNAH5	1.89	5.77 × 10^6^	0.010	CARD11	−1.72	1.49 × 10^4^	0.032
SV2C	1.88	1.05 × 10^4^	0.027	GBP5	−1.68	3.74 × 10^5^	0.018
COL11A1	1.82	1.63 × 10^6^	0.004	DUSP2	−1.61	2.63 × 10^6^	0.006
PPFIA2	1.70	6.51 × 10^6^	0.010	LLGL1	−1.47	2.81 × 10^4^	0.044
COL21A1	1.69	3.55 × 10^5^	0.018	CD52	−1.42	1.07 × 10^4^	0.027
COL11A2	1.68	9.38 × 10^6^	0.010	CD72	−1.31	1.22 × 10^4^	0.029
CRACD	1.63	7.48 × 10^5^	0.023	TAP2	−1.29	6.97 × 10^5^	0.023
RBFOX3	1.55	1.71 × 10^5^	0.013	FOLR2	−1.26	3.35 × 10^4^	0.049
MYB	1.49	1.21 × 10^7^	0.001	LCP2	−1.25	3.59 × 10^4^	0.050
MECOM	1.48	2.02 × 10^5^	0.014	NLRC5	−1.24	5.69 × 10^5^	0.022
TCEA3	1.41	9.54 × 10^5^	0.025	SPN	−1.19	3.22 × 10^4^	0.048
MLF1	1.41	8.13 × 10^6^	0.010	SASH3	−1.16	1.61 × 10^4^	0.033
VSIG8	1.40	2.33 × 10^4^	0.041	C1QB	−1.10	2.19 × 10^4^	0.040
MDGA1	1.40	6.61 × 10^5^	0.023	B4GALNT4	−1.06	3.15 × 10^4^	0.047
H2AC8	1.39	4.70 × 10^5^	0.021	HLA-DRA	−1.05	3.05 × 10^5^	0.017
TRIM2	1.39	8.08 × 10^5^	0.023	FMC1	−1.01	2.21 × 10^4^	0.040
H1-3	1.35	2.46 × 10^4^	0.042	RFT1	−1.01	1.38 × 10^5^	0.012
MELTF	1.33	7.17 × 10^5^	0.023	CD74	−1.01	1.93 × 10^4^	0.036
DST	1.30	7.70 × 10^7^	0.004	LDLRAD3	−0.92	2.79 × 10^4^	0.044
PHLDA1	1.29	7.98 × 10^5^	0.023	CIB1	−0.85	3.51 × 10^4^	0.050
ENC1	1.29	1.25 × 10^4^	0.029	HLA-E	−0.72	6.14 × 10^5^	0.022
SYT11	1.28	1.33 × 10^5^	0.012	ATF6B	−0.72	2.38 × 10^4^	0.041
XYLT1	1.25	1.18 × 10^4^	0.029	PSME1	−0.65	1.57 × 10^4^	0.033
VEGFA	1.25	1.29 × 10^4^	0.029	RPS4X	−0.65	5.36 × 10^5^	0.022
CADM1	1.23	3.66 × 10^5^	0.018	MXD4	−0.63	7.98 × 10^5^	0.023
NTNG2	1.13	8.34 × 10^5^	0.023	GNAI2	−0.56	1.55 × 10^4^	0.033
NEDD4L	1.13	7.85 × 10^5^	0.023	OGFR	−0.48	3.13 × 10^4^	0.047
SPATA6	1.10	2.32 × 10^4^	0.041				
RRBP1	1.09	1.43 × 10^6^	0.004				
KIF23	1.02	2.66 × 10^4^	0.043				
SULF2	1.01	1.41 × 10^4^	0.031				
CCDC30	1.00	1.83 × 10^4^	0.035				
ITGA6	0.99	3.42 × 10^4^	0.049				
PCDH9	0.98	2.96 × 10^4^	0.046				
SMYD3	0.97	1.27 × 10^4^	0.029				
IGFBP5	0.96	5.95 × 10^5^	0.022				
ADAM12	0.93	2.54 × 10^4^	0.042				
CIT	0.93	8.85 × 10^5^	0.024				
MEIS2	0.91	1.42 × 10^5^	0.012				
ASB1	0.84	4.80 × 10^5^	0.021				
ATN1	0.83	1.75 × 10^4^	0.034				
TUBA1A	0.81	3.14 × 10^5^	0.017				
AKT3	0.80	3.05 × 10^5^	0.017				
TMCO3	0.78	9.69 × 10^5^	0.025				
CADM4	0.71	1.72 × 10^4^	0.034				
TPM1	0.66	3.57 × 10^4^	0.050				
CHD7	0.65	1.75 × 10^4^	0.034				
HP1BP3	0.56	2.56 × 10^4^	0.042				

**Table 4:** Gene Ontology (GO) terms most significantly enriched by death-associated genes.

Top 10 GO Biological Process Terms Most Significantly Enriched by Death-Associated Genes			
GO Term	*p*-Value	q-Value	Genes
chordate embryonic development (GO:0043009)	3.30 × 10^7^	<0.001	[CHD7, IGF2, XYLT1, VEGFA, SULF2]
collagen fibril organization (GO:0030199)	2.82 × 10^6^	0.001	[DST, COL11A1, COL11A2, COL21A1, ITGA6]
supramolecular fiber organization (GO:0097435)	2.52 × 10^5^	0.006	[DST, COL11A1, TPM1, COL11A2, TCHH, COL21A1, ITGA6]
in utero embryonic development (GO:0001701)	3.25 × 10^5^	0.006	[CHD7, IGF2, VEGFA]
skeletal system development (GO:0001501)	4.59 × 10^5^	0.006	[CHD7, COL11A2, IGF2, XYLT1, SULF2]
extracellular matrix organization (GO:0030198)	9.89 × 10^5^	0.011	[DST, COL11A1, ADAM12, COL11A2, COL21A1, ITGA6]
regulation of vascular associated smooth muscle cell migration (GO:1904752)	3.98 × 10^4^	0.034	[IGFBP5, TPM1]
hemidesmosome assembly (GO:0031581)	3.98 × 10^4^	0.034	[DST, ITGA6]
positive regulation of vascular endothelial cell proliferation (GO:1905564)	4.69 × 10^4^	0.034	[AKT3, IGF2]
heterochromatin organization (GO:0070828)	5.47 × 10^4^	0.034	[MECOM, HP1BP3]
**Top 10 GO Molecular Function Terms Most Significantly Enriched by Death-Associated Genes**			
vascular endothelial growth factor receptor 2 binding (GO:0043184)	1.70 × 10^4^	0.012	[CADM4, VEGFA]
vascular endothelial growth factor receptor binding (GO:0005172)	3.98 × 104	0.012	[CADM4, VEGFA]
insulin-like growth factor I binding (GO:0031994)	4.69 × 10^4^	0.012	[IGFBP5, ITGA6]
insulin-like growth factor binding (GO:0005520)	6.30 × 10^4^	0.012	[IGFBP5, ITGA6]
histone-lysine N-methyltransferase activity (GO:0018024)	5.66 × 10^3^	0.087	[MECOM, SMYD3]
PDZ domain binding (GO:0030165)	1.09 × 10^2^	0.137	[CADM1, CIT]
neuregulin binding (GO:0038132)	1.24 × 10^2^	0.137	[ITGA6]
myosin light chain binding (GO:0032027)	1.49 × 10^2^	0.140	[SPATA6]
insulin-like growth factor II binding (GO:0031995)	1.74 × 10^2^	0.140	[IGFBP5]
sodium channel inhibitor activity (GO:0019871)	1.98 × 10^2^	0.140	[NEDD4L]

**Table 5:** Gene Ontology (GO) terms most significantly enriched by survival-associated genes.

Top 10 GO Biological Process Terms Most Significantly Enriched by Survival-Associated Genes			
GO Term	*p*-Value	q-Value	Genes
antigen processing and presentation of endogenous peptide antigen (GO:0002483)	9.87 × 10^7^	<0.001	[TAP2, HLA-DRA, HLA-E]
positive regulation of immune response (GO:0050778)	4.03 × 10^6^	0.001	[SASH3, CD74, GBP5, HLA-DRA]
positive regulation of innate immune response (GO:0045089)	1.89 × 10^5^	0.003	[GBP5, NLRC5, HLA-E]
T cell receptor signaling pathway (GO:0050852)	7.63 × 10^5^	0.009	[PSME1, HLA-DRA, LCP2, CARD11]
antigen receptor-mediated signaling pathway (GO:0050851)	1.40 × 10^4^	0.012	[PSME1, HLA-DRA, LCP2, CARD11]
antigen processing and presentation of exogenous peptide antigen via MHC class I, TAP-dependent (GO:0002479)	1.59 × 10^4^	0.012	[TAP2, PSME1, HLA-E]
positive regulation of lymphocyte proliferation (GO:0050671)	1.73 × 10^4^	0.012	[SASH3, CD74, HLA-E]
antigen processing and presentation of exogenous peptide antigen via MHC class I (GO:0042590)	1.94 × 10^4^	0.012	[TAP2, PSME1, HLA-E]
positive regulation of alpha-beta T cell activation (GO:0046635)	3.80 × 10^4^	0.020	[HLA-DRA, HLA-E]
antigen processing and presentation of exogenous peptide antigen (GO:0002478)	4.40 × 10^4^	0.021	[CD74, HLA-DRA, HLA-E]
**Top 10 GO Molecular Function Terms Most Significantly Enriched by Survival-Associated Genes**			
MHC protein binding (GO:0042287)	9.00 × 10^6^	0.001	[CD74, TAP2, HLA-E]
MHC class I protein binding (GO:0042288)	2.72 × 10^4^	0.005	[TAP2, HLA-E]
MHC class II protein complex binding (GO:0023026)	2.72 × 10^4^	0.005	[CD74, HLA-DRA]
TAP1 binding (GO:0046978)	7.23 × 10^3^	0.061	[TAP2]
MHC class II protein binding (GO:0042289)	8.67 × 10^3^	0.061	[CD74]
peptide transmembrane transporter activity (GO:1904680)	1.01 × 10^2^	0.061	[TAP2]
MHC class Ib protein binding (GO:0023029)	1.15 × 10^2^	0.061	[TAP2]
CD4 receptor binding (GO:0042609)	1.15 × 10^2^	0.061	[CD74]
natural killer cell lectin-like receptor binding (GO:0046703)	1.30 × 10^2^	0.061	[HLA-E]
guanylate kinase activity (GO:0004385)	1.30 × 10^2^	0.061	[CARD11]

**Table 6:** Most significantly enriched Kyoto Encyclopedia of Genes and Genomes (KEGG) pathways.

Top 10 KEGG Pathways Most Significantly Enriched by Death-Associated Genes			
KEGG Pathway Term	*p*-Value	q-Value	Genes
PI3K-Akt signaling pathway	1.86 × 10^3^	0.138	[MYB, AKT3, IGF2, ITGA6, VEGFA]
Protein digestion and absorption	2.18 × 10^3^	0.138	[COL11A1, COL11A2, COL21A1]
Cell adhesion molecules	6.04 × 10^3^	0.164	[NTNG2, CADM1, ITGA6]
MAPK signaling pathway	6.20 × 10^3^	0.164	[MECOM, AKT3, IGF2, VEGFA]
VEGF signaling pathway	9.57 × 10^3^	0.164	[AKT3, VEGFA]
Pathways in cancer	1.03 × 10^2^	0.164	[MECOM, AKT3, IGF2, ITGA6, VEGFA]
Lysine degradation	1.09 × 10^2^	0.164	[MECOM, SMYD3]
Renal cell carcinoma	1.29 × 10^2^	0.164	[AKT3, VEGFA]
Focal adhesion	1.39 × 10^2^	0.164	[AKT3, ITGA6, VEGFA]
Proteoglycans in cancer	1.46 × 10^2^	0.164	[AKT3, IGF2, VEGFA]
**Top 10 KEGG Pathways Most Significantly Enriched by Survival-Associated Genes**			
Antigen processing and presentation	8.74 × 10^8^	<0.001	[CD74, TAP2, PSME1, HLA-DRA, HLA-E]
Human cytomegalovirus infection	2.97 × 10^4^	0.015	[ATF6B, TAP2, HLA-E, GNAI2]
Cell adhesion molecules	1.26 × 10^3^	0.025	[SPN, HLA-DRA, HLA-E]
Phagosome	1.36 × 10^3^	0.025	[TAP2, HLA-DRA, HLA-E]
Allograft rejection	1.38 × 10^3^	0.025	[HLA-DRA, HLA-E]
Graft-versus-host disease	1.69 × 10^3^	0.025	[HLA-DRA, HLA-E]
Type I diabetes mellitus	1.77 × 10^3^	0.025	[HLA-DRA, HLA-E]
Cocaine addiction	2.29 × 10^3^	0.029	[ATF6B, GNAI2]
Autoimmune thyroid disease	2.67 × 10^3^	0.029	[HLA-DRA, HLA-E]
Epstein–Barr virus infection	3.06 × 10^3^	0.029	[TAP2, HLA-DRA, HLA-E]

## Data Availability

Publicly available datasets were analyzed in this study. These data can be found at PRJNA775071.

## Supplementary Materials

The following supporting information can be downloaded at https://www.mdpi.com/article/10.3390/cancers14153591/s1. Table S1: Gene Ontology Biological Process, Gene Ontology Molecular Function, and Kyoto Encyclopedia of Genes and Genomes pathway terms significantly enriched by death-associated genes; Table S2: Gene Ontology Biological Process, Gene Ontology Molecular Function, and Kyoto Encyclopedia of Genes and Genomes pathway terms significantly enriched by survival-associated genes; Table S3: Genes associated with disease specific death and survival when using ≥3 years survival as a criterion for inclusion in the good prognosis group; Table S4: Gene Ontology Biological Process, Gene Ontology Molecular Function, and Kyoto Encyclopedia of Genes and Genomes pathway terms significantly enriched by survival-associated genes when using ≥3 years survival as a criterion for inclusion in the good prognosis group; Table S5: Gene Ontology Biological Process, Gene Ontology Molecular Function, and Kyoto Encyclopedia of Genes and Genomes pathway terms significantly enriched by death-associated genes when using ≥3 years survival as a criterion for inclusion in the good prognosis group; Table S6: Genes significantly upregulated in MCPyV-negative tumors, as compared with MCPyV-positive tumors, of patients who died from MCC; Table S7: Pearson’s product–moment correlation between the abundance of CD3-positive, tumor-infiltrating lymphocytes and the expression levels of the genes CD74, TAP2, PSME1, HLA-DRA, and HLA-E; Table S8: List of the 136/250 genes most significantly differentially expressed between MCPyV-positive and -negative tumors in GSE39612, which were also detectable in FFPE data.
